# Fertility-sparing surgery and oncologic outcome among patients with early-stage ovarian cancer ~propensity score- matched analysis~

**DOI:** 10.1186/s12885-019-6432-4

**Published:** 2019-12-19

**Authors:** Hiroaki Kajiyama, Shiro Suzuki, Nobuhisa Yoshikawa, Michiyasu Kawai, Kimio Mizuno, Osamu Yamamuro, Tetsuro Nagasaka, Kiyosumi Shibata, Fumitaka Kikkawa

**Affiliations:** 1 Department of Obstetrics and Gynecology., Graduate School of Medicine, Nagoya University, Truma-cho 65, Showa-ku, Nagoya, 466-8550 Japan; 2 Department of Obstetrics and Gynecology, Toyohashi Municipal Hospital, Toyohashi, Japan; 3 Department of Department of Obstetrics and Gynecology, Nagoya First Red-cross Hospital, Nagoya, Japan; 4 Department of Obstetrics and Gynecology, Nagoya Second Red-cross Hospital, Nagoya, Japan; 5 Division of Medical Laboratory Sciences, School of Health Science, Nagoya University, Nagoya, Japan; 6 Department of Obstetrics and Gynecology, Bantane Hospital, Fujita Health University, Nagoya, Japan

**Keywords:** Epithelial ovarian carcinoma, Fertility-sparing surgery, Overall survival, Recurrence, Recurrence-free survival, Propensity score

## Abstract

**Background:**

The aim of this study was to investigate how much the risks of recurrence and death are increased as a consequence of selecting fertility-sparing surgery (FSS) in young women with epithelial ovarian cancer (EOC).

**Methods:**

After a central pathological review and search of the medical records from 14 collaborating hospitals, a non-randomized, observational cohort study was conducted between 1987 and 2015, including 1183 women with stage I EOC. Finally, a total of 285 patients with stage I EOC at reproductive age were recruited. Oncologic outcomes were compared between the FSS (*N* = 101) and radical surgery (RS) group (*N* = 184) using a propensity score (PS)-matching technique to adjust for relevant risk factors: the age, substage, histological type, grade, CA125 values, ascites cytology, ascites volume, and chemotherapy.

**Results:**

During 66.0 months (median) of follow-up, 42 patients (14.7%) developed recurrence, and 31 patients (10.9%) died. In the original cohort, there was no significant difference in overall survival (OS) or recurrence-free survival (RFS) between the FSS and RS groups {Log-rank: OS (*P* = 0.838), RFS (*P* = 0.377)}. In the PS-matched cohort after adjustment for multiple clinicopathologic factors, there was no significant difference in RFS or OS between the FSS and RS groups {RFS (FSS vs. RS), HR: 1.262 (95% CI: 0.559–2.852), *P* = 0. 575; OS (FSS vs. RS), HR: 1.206 (95% CI: 0.460–3.163), *P* = 0.704}.

**Conclusions:**

After adjustment for clinicopathologic factors, FSS in itself may not worsen the oncologic outcome in young women with early-stage EOC. A large-scale clinical study is necessary to validate the findings.

## Background

Epithelial ovarian cancer (EOC) is the one of the most lethal cancers among gynecologic malignancies worldwide, with more than 238,700 newly diagnosed cases and 151,900 reported deaths per year [1]. In general, this tumor is common in postmenopausal women. However, based on several prior studies, 3–17% of patients with EOC are of reproductive age: under or around 40 years of age [2–6]. If we select conventional surgical procedures in such reproductive-age patients, female-specific endocrine and reproductive functions will be lost. Needless to say, it is most important for us to aim for the complete cure of those women with early-stage EOC. Nevertheless, conserving such a function is also crucial for maintaining their quality of life.

Usually, fertility-sparing surgery (FSS) has been acceptably chosen for young patients with an ovarian-confined / capsulated / well-differentiated EOC. Unfortunately, we cannot accurately estimate rate of recurrence and subsequent mortality will be increased in patients receiving FSS compared with radical surgery, reflecting the difficulty of performing a randomized controlled trial. Several researchers have attempted to determinate the long-term effectiveness of FSS by comparing the oncologic outcome between two cohorts [7, 8]. Nevertheless, it is difficult to conduct a simple comparison because there are many biases between them, including the substage, tumor differentiation, histological type, and presence or absence of chemotherapy.

Recently, there has been increasing interest in applying a propensity score methodology to reduce or eliminate the effects of confounding when analyzing observational data. In the current study, we investigated the impact of FSS on recurrence-free and overall survival in young patients with early-stage EOC in a multicentric analysis using a propensity score-matching technique.

## Methods

### Patient enrollment

Between January/1987 and December/2015, 4237 patients with malignant ovarian tumors were registered and accumulated by the Tokai Ovarian Tumor Study Group (TOTSG), consisting of 14 collaborating institutions [9]. All histological slides were reviewed by two expert pathologists with no knowledge of the patients’ clinical data under a central pathological review system. Eligible cases included: 1) age under 45 years old at the time of the initial diagnosis, 2) histologically confirmed stage I EOC, 3) received initial surgery and periodic follow-up at the aforementioned institutions. Accordingly, of these, there were 1183 patients with stage I EOC with sufficient clinical information. Consequently, from this database, 285 patients aged younger than or equal to 45 years who had a stage I EOC were analyzed, including 101 patients who had received FSS and 184 who had undergone radical surgery (RS) (Additional file 2: Figure. S1). As the histological types, we adopted the World Health Organization (WHO) classification criteria. The stage was assigned according to the International Federation of Gynecology and Obstetrics (FIGO) staging system [10, 11]. This study was approved by the ethics committee of Nagoya University.

### Treatments

The standard surgeries in patients who belonged to the RS cohort were in principal hysterectomy and bilateral salpingo-oophorectomy with the complete staging surgery. The complete staging surgery was defined as lymph node evaluation and peritoneal staging. The peritoneal staging included cytology of ascites or washing, and/or omentectomy (or biopsy), and appropriate peritoneal biopsy if necessary. Lymph node evaluation involved one of the following: 1) lymph node sampling, 2) lymph node dissection, or 3) palpation and removal of enlarged lymph nodes. The selecting principles in women who received the FSS were as follows: 1) Women had strongly desired to preserve fertility, 2) those were informed of the possible benefits and risks of FSS, and signed a consent form in a preoperative counseling session. The surgical approach for these patients were at least conservation of the contralateral ovary and uterus with a full peritoneal staging. Omentectomy, wedge resection of the remaining ovary, and systematic retroperitoneal lymphadenectomy were optional. However, the absence of an enlarged lymph node more than 1 cm in diameter was confirmed by preoperative imaging; if present, enlarged nodes were appropriately sampled [9].

Of all stage I patients, 214 were treated postoperatively with 3 to 6 cycles of adjuvant platinum-based chemotherapy. A total of 71 patients (24.9%) did not receive adjuvant platinum-based chemotherapy due to severe complications, the patients’ wishes, meeting the criterion of omission (stage IA/grade 1–2), or the decision of each institution. Details of the chemotherapy regimen in each period were as follows: CAP [cyclophosphamide (300 mg/m^2^), adriamycin (30 mg/m^2^), and cisplatin (70 mg/m^2^)] (1986–1989); CAP or PVB [cisplatin (70 mg/m^2^), vinblastine (6 mg/m^2^), and bleomycin (12 mg/m^2^)] (1989–1991); PVB or PP [carboplatin (300 mg/m^2^) and cisplatin (70 mg/m^2^)] (1992–2000); TC {paclitaxel (180 mg/m^2^) and carboplatin [area under curve (AUC = 5–6]} (2000–2002); TC or DC [docetaxel (70 mg/m^2^) and carboplatin (AUC 5–6)] (2003–2013); TC or DC with or without bevacizumab (15 mg/kg) (2013–) [12].

### Follow-up and analysis

All patients received a thorough follow-up and periodic checkups, including gynecologic examination, CA125 evaluation, ultrasonography, and radiologic imaging based on the Gynecologic Cancer InterGroup (GCIG) criteria [9, 13]. The recurrence-free survival (RFS) was defined as the time interval between the date of surgery and that of recurrence or the last follow-up. The overall survival (OS) was defined as the time between the date of surgery and that of the last follow-up or death from any cause. The distributions of clinicopathologic events were evaluated using the Chi-square tests. To balance the patient and tumor characteristics between FSS and RS groups, propensity score (PS) matching was performed [14]. PS was estimated by multivariate logistic regression models for the probability of FSS adjusting for age, FIGO stage, histological type, tumor grade, preoperative CA125 value, ascites volume, cytology of ascites, and presence or absence of chemotherapy. Patients with FSS were matched with RS counterparts according to PS, leading to an even distribution of potential confounding factors in both groups. Within the original and PS-matched cohort, survival curves were generated using Kaplan-Meier methods. A Cox proportional hazards regression model was used to examine associations between the type of surgery (FSS vs. RS) and RFS/OS. All statistical analyses were performed with SPSS Ver. 26 (IBM Japan, Tokyo) and JMP Pro Ver.10.0 (SAS Institute Japan). A *P*-value of < 0.05 was considered significant.

## Results

### Patients’ characteristics

In total, 285 women were identified for the current analysis. Patients’ characteristics are shown in Table 1. The cohort included 101 women (35.4%) who underwent FSS and 184 women (64.6%) who had RS. The median (SD) age of those who received FSS was 33 (7.6) years. Patients who underwent FSS were significantly younger than were those who received RS (*P* < 0.0001) (Table 1). The median follow-up duration of all patients was 66.0 months. There was no difference in the follow-up duration between the FSS group (median: 62.6 months) and RS group (68.7 months) (*P* = 0.296). Regarding the distribution of the substage, preoperative CA125 value, volume of ascites, and ascites cytology, there was no difference between the two groups. With regard to histological types, a clear-cell histology was more frequently observed in the RS group than in the FSS group (*P* < 0.0001). In addition, adjuvant chemotherapy was more frequently conducted in the RS group than in the FSS group (*P* = 0.0007).

**Table 1:** Patients’ characteristics

	RS		FSS		*P*-value*
	N	%	N	%	
Total	184		101		
Age (median/mean/SD)	41/40.3/4.3		33/32.0/7.6		< 0.0001
FIGO stage					
IA	66	35.9	43	42.6	0.3004^#1^
IB	2	1.1	0	0.0	
IC1	74	40.2	43	42.6	
IC2/IC3	42	22.8	15	14.9	
Histological type					
Clear-cell	73	39.7	22	21.8	< 0.0001
Mucinous	38	20.7	51	50.5	
Endometrioid	54	29.3	24	23.8	
Serous	15	8.2	3	3.0	
Mix	3	1.6	0	0.0	
Others^#1^	1	0.5	1	1.0	
Grade					
G1/G2	106	57.6	79	78.2	0.0005
G3/Clear-cell	78	42.4	22	21.8	
CA125					
≤ 35 U/mL	71	38.6	48	47.5	0.143
> 35 U/mL	113	61.4	53	52.5	
Ascites volume					
≤ 100 mL	156	84.8	87	86.1	0.757
> 100 mL	28	15.2	14	13.9	
Ascites cytology					
Negative	159	86.4	94	93.1	0.0887
Positive	25	13.6	7	6.9	
Chemotherapy					
Absent	34	18.5	37	36.6	0.0007
Present	150	81.5	64	63.4	

*FIGO* Internatinal Federation of Gynecology and Obstetrics, #1: IA vs. IB vs. IC

### Oncologic outcome using the original cohort

**Fig. 1:**
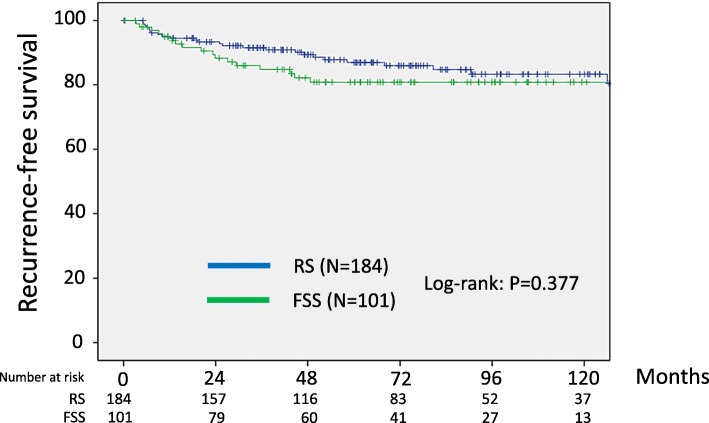
Kaplan-Meier-estimated recurrence-free survival (RFS) on stratifying by the surgical type {FSS (*N* = 101) vs. RS (*N* = 184)}. The original cohort

**Fig. 2:**
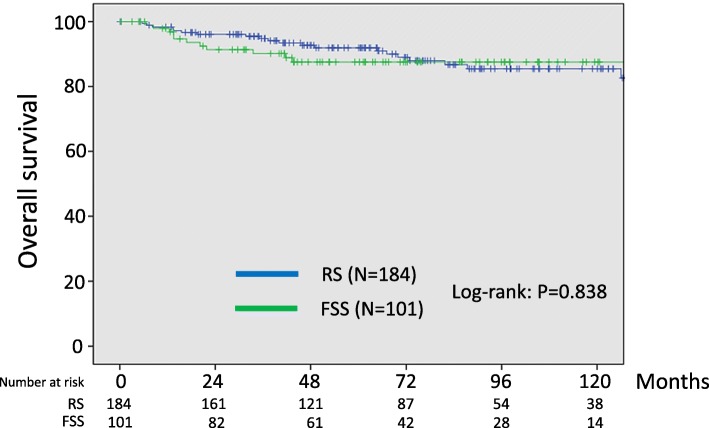
Kaplan-Meier-estimated overall survival (OS) on stratifying by the surgical type {FSS (*N* = 101) vs. RS (*N* = 184)}. The original cohort

With follow-up of a total of 285 patients, 42 patients (14.7%) developed recurrence. In addition, 31 patients (10.9%) died of the disease. Recurrent disease was noted in 17 (16.8%) patients in the FSS group and 25 (13.6%) patients in the RS group. Death was noted in 11 (10.9%) patients in the FSS group and 20 (10.9%) patients in the RS group. In the original cohort, the 5-year recurrence-free survival rates (95% CI) of the FSS and RS groups were 80.8 (71.1–87.8)% and 86.9 (80.6–91.4)%, respectively. As a result, we did not identify any significant difference between the two groups (Log-rank: *P* = 0.377) (Fig.1). In addition, the 5-year overall survival rates (95% CI) of the FSS and RS group were 87.5 (78.8–93.0)% and 91.9 (86.5–95.3)%, respectively. Also, there was no significant difference between the two groups (Log-rank: *P* = 0.838) (Fig.2).

### Oncologic outcome using the PS-matched cohort

**Fig. 3:**
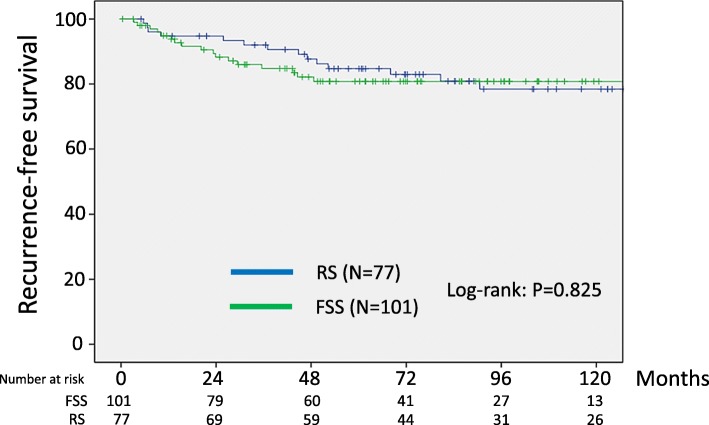
Kaplan-Meier-estimated RFS on stratifying by the surgical type {FSS (*N* = 101) vs. RS (*N* = 77)}. The PS-matched cohort. Calculation of PS was then performed for each patient based on eight clinicopathologic variables, including the age, substage, histological type, grade, volume of ascites, ascites cytology, presence or absence of chemotherapy

**Fig. 4:**
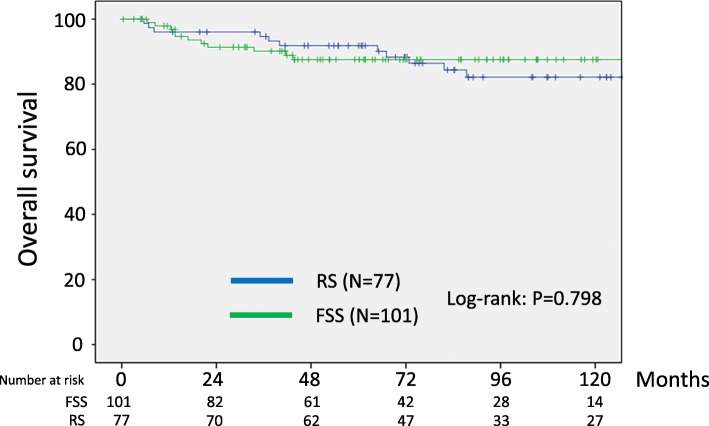
Kaplan-Meier-estimated OS on stratifying by the surgical type {FSS (N = 101) vs. RS (N = 77)}. The PS-matched cohort

Calculation of PS was then performed for each patient based on eight clinicopathologic variables: the age, substage, histological type, grade, volume of ascites, CA125 value, cytology, and presence or absence of chemotherapy. The reasons why these conditioning variables were selected were that they were relevant to survival but were not balanced in either the FSS or RS setting. Consequently, 178 matched pairs were generated using PS-matching. Additional file 1: Table S3 summarizes patients’ characteristics after matching. After PS-matching, all conditioning variables except for the age and performance of chemotherapy were well-balanced (Additional file 1: Table S3). In the PS-matching cohort, the 5-year RFS (95% CI) rate was 80.8 (71.1–87.8)% for the FSS group and 84.7 (74.4–91.3)% for the RS group (Log-rank: *P* = 0.825) (Fig.3). In addition, the 5-year overall survival rates were 87.5 and 91.8% in patients with FSS and RS, respectively (Fig.4). The difference was also non-significant between the two surgical groups (Log-rank: *P* = 0.798). Thus, after the PS-matching, FSS and OS maintained the similar trends with the full dataset.

Table S1 summarizes the results of a multivariable Cox hazard model for recurrence outcomes using the original cohort data. In a crude analysis, there was no association between the surgical type and RFS {HR (95% CI): 1.319 (0.712–2.442), *P* = 0.379}. Even after adjusting for several combinations of clinicopathologic confounders listed, we did not identify any significant prognostic correlation between the surgical type and RFS {adjusted by multi-factors: HR (95% CI): 1.543 (0.722–3.297), *P* = 0.263}. Similarly, there was no significant prognostic correlation between the performance of FSS and OS, even after adjusting for several combinations of clinicopathologic confounders {adjusted by age, sub-stage, grade, CA125 value, ascites cytology, and chemotherapy: HR (95% CI): 1.763 (0.725–4.288), *P* = 0.211} (Additional file 1: Table S2).

In Cox multivariable hazard model, after adjustment for multiple confounders, including the age, PS, surgical type, substage, grade, CA125 value, ascites cytology, and chemotherapy, the performance of FSS itself was not a significant predictor of the risk of recurrence {adjusted HR (95% CI): 1.262 (0.559–2.852), *P* = 0.575} (Additional file 1: Table S4). Similarly, in multivariable analyses for OS, the same tendencies were observed {adjusted HR (95% CI): 1.206 (0.460–3.163), *P* = 0.704} (Table 2).

**Table 2:** Cox Proportional Hazards Analyses of OS among patients who underwent FSS

Propensity-Matched patients			
Model	Hazard Ratio	95% CI	*P*-value
Unadjusted	0.897	0.388–2.072	0.799
Adjusted for PS	1.054	0.398–2.789	0.916
Adjusted for PS, age, sub-stage^#1^, and ascites volume	1.281	0.461–3.556	0.635
Adjusted for PS and multi-factors^#2^	1.206	0.460–3.163	0.704

*OS* overall survival, *FSS* fertility-sparing surgery, *PS* propensity score, #1: IA/IB/IC1 vs. IC2/IC3, #2: surgery, age, substage, grade, CA125 value, ascites cytology, and chemotherapy

## Discussion

A woman who selects FSS receives the benefits of preserving the possibility of having a child in the future, regardless of the risk of unexpected recurrence. When we consider whether FSS should be selected for a woman with early-stage EOC at reproductive age, we subconsciously fear the risk of a future recurrence or subsequent death from disease. Here, we encounter the fundamental question of how much the preservation of the contralateral ovary and uterus is associated with the recurrence. On considering clinical information on the extent that recurrence is increased or how different long-term survival is between patients with FSS and those receiving radical surgery, it is beneficial for the patient and physician to share risk-and-benefit data before selecting this surgery. The randomized controlled trial (RCT) is a solution to this problem, but it is actually very difficult to perform for ethical reasons. In our earlier study, we preliminarily reported that the 5-year overall survival rates in the three groups of patients with stage I EOC were 90.8% (FSS at reproductive age), 88.3% (non-FSS at reproductive age), and 90.6% (non-FSS in the elderly), concluding that there was no significant difference on three-group comparison [15]. Since then, several retrospective studies have demonstrated similar results, suggesting the non-inferiority of the long-term outcome in patients who underwent FSS, compared with those received conventional surgery [7, 8]. Nevertheless, these investigations had a critical limitation associated with any retrospective study, involving the possibility of selection bias and treatment heterogeneity. Even if showing a non-significant difference in oncologic outcomes, a number of clinicopathological profiles were inconsistent between the two cohorts. At least, considering major clinical backgrounds of patients with stage I EOC, the three categories of “substage”, “degree of differentiation”, and “histological type” overlap with one another and are complicated. For example, we can easily expect that patients with favorable clinicopathological factors, including an encapsulated, well-differentiated, chemosensitive histological type will tend to undergo FSS. Thus, considering this underlying bias, the results showing no difference in the oncologic outcome may erroneously suggest that FSS has a negative effect on survival. An RCT is actually very difficult to perform because of ethical problems. PS-matching is an efficient methodology to reduce bias by balancing many measured confounders between treatment and control groups. Recently, abundant evidence revealed the usefulness of a PS-matching technique mimicking some aspects of an RCT [14, 16–19]. In the present study, to assess the appropriateness of FSS, we compared the survival between larger groups of patients who had undergone FSS and those who had received non-FSS radical surgery using the original and PS-matching cohorts. Consequently, the comparison between the two surgical groups revealed no difference in recurrence-free or overall survival rates. Thus, the current PS-matching study provides evidence that the implementation of FSS does not necessarily lead to lower progression-free and overall survival than conventional non-FSS surgery. Furthermore, we showed the therapeutic efficacy of FSS in the treatment of early-stage EOC at reproductive age. Taken together, FSS is worthy of consideration for young patients diagnosed with early-stage EOC.

Our current work still includes several limitations. Initially, because the present study was essentially a retrospective study, many factors relevant to the treatment decision were not as strictly controlled as they would be in an RCT. Particularly, our PS-matching model was still not balanced for the age and receipt of adjuvant chemotherapy. Subsequently, the composition of the study subjects may have been influenced by referral bias owing to its multicentric design for a long-term study period. Lastly, several critical data, such as socioeconomic profiles, were not provided, which may affect the reliability of the estimated PS. In contrast, the strengths of our study: firstly, the performance of central pathological review by expert pathologists for gynecologic malignancy; secondly, the relatively high patient number; and thirdly, the same chemotherapeutic criteria and protocol as for the identical study group (TOTSG group).

## Conclusion

In summary, we examined the fundamental question of how much the preservation of the contralateral ovary and uterus is associated with recurrence. On considering a clinical information on the extent that recurrence is increased or how different long-term survival is between patients with FSS and those receiving radical surgery, it is beneficial for the patient and physician to share risk-and-benefit data before selecting this surgery. On this occasion, we merely put forward a hypothesis that patients with stage I EOC who have undergone FSS may not show a poorer prognosis than those receiving radical surgery. Concerning the patients’ specificity and ethical consideration, an RCT is unlikely from now on. In the present study, we included women aged in their early 40s. With the progression of the trends of late marriage and a low birthrate, we will more frequently encounter this demographic in our daily clinical practice. The number of women over 40 years of age seeking infertility treatment has been steadily increasing [20]. Actually, the percentage of women in their early 40s requiring assisted reproductive technology has increased significantly from 10 to 15% in the early 2000s to 20 to 25% in 2009 [20–22]. Thus, we should reassess the possibility of FSS based on a larger number of patients, including those in their 40s. Taken together, we should accumulate further cases to clarify treatment prospects. We hope that the hypothesis will be supported by accumulating more patients treated with FSS through a large-scale clinical registry system developed in the near future.

## Supplementary information

**Additional file 1: Table S1.** Cox Proportional Hazards Analyses of RFS among patients who underwent FSS (Original cohort), **Table S2.** Cox Proportional Hazards Analyses of OS among patients who underwent FSS (Original cohort), **Table S3.** Patients’ characteristics (PS-matching), **Table S4.** Cox Proportional Hazards Analyses of RFS among patients who underwent FSS.

**Additional file 2: Figure S1.** Patient flowchart.

## Data Availability

The datasets generated and/or analyzed during the current study are not publicly available due owing to data privacy policy at our facility, but are available from the corresponding author on reasonable request.

## Abbreviations

EOC: Epithelial ovarian carcinoma
FSS: Fertility-sparing surgery
OS: Overall survival
PS: Propensity score
RFS: Recurrence-free survival
RS: Radical surgery
