# Supplementary material for: Fertility-sparing surgery and oncologic outcome among patients with early-stage ovarian cancer ~propensity score- matched analysis~
Source: BMC Cancer. 2019 Dec 19;19:1235. doi: 10.1186/s12885-019-6432-4 (PMC6921416; doi:10.1186/s12885-019-6432-4)
Supplement: Supplementary file 2 — Additional file 2: Figure S1. Patient flowchart. [file 12885_2019_6432_MOESM2_ESM.pptx]

## Slide 1
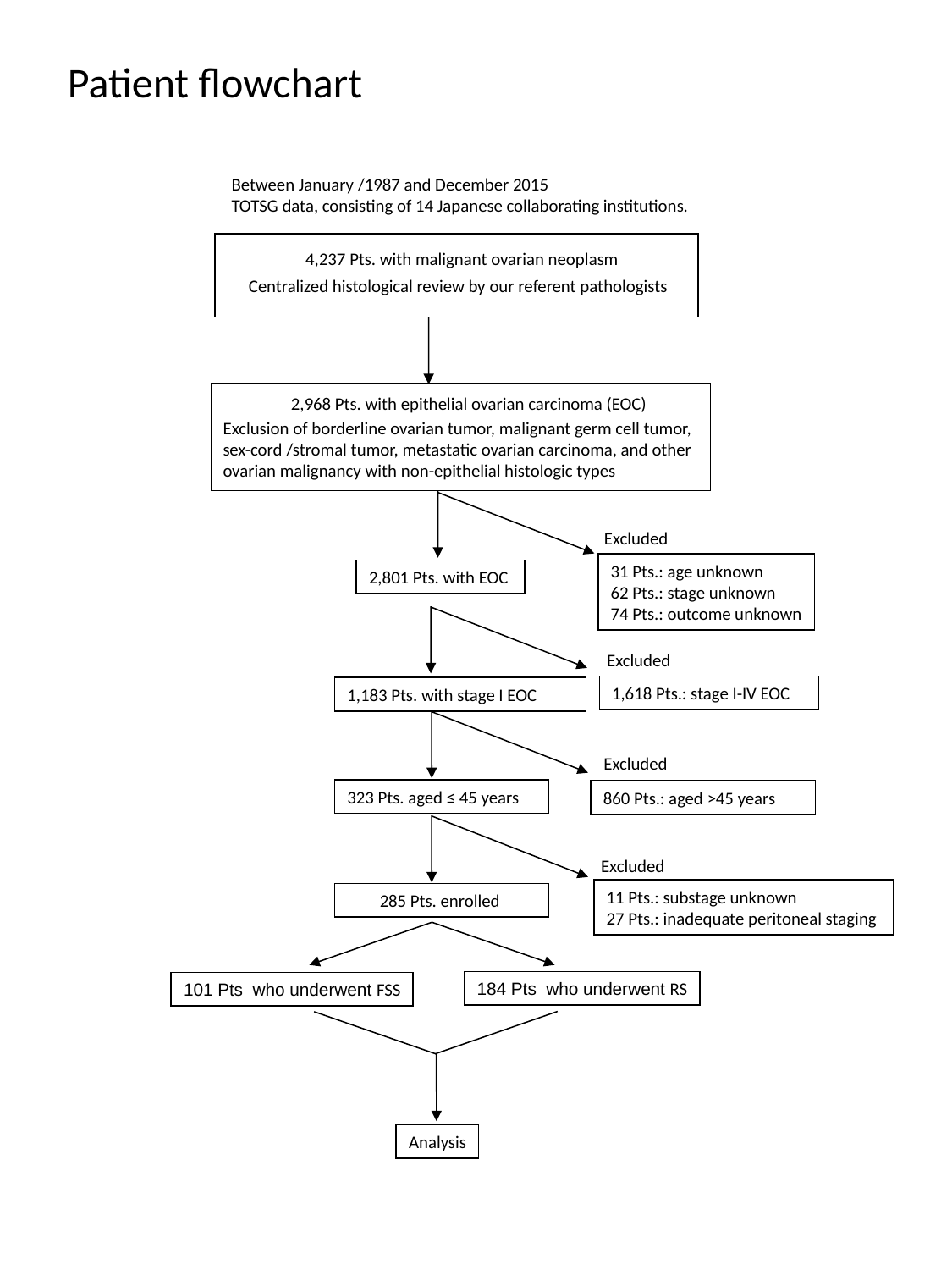

Patient flowchart
Between January /1987 and December 2015
TOTSG data, consisting of 14 Japanese collaborating institutions.
4,237 Pts. with malignant ovarian neoplasm
Centralized histological review by our referent pathologists
2,968 Pts. with epithelial ovarian carcinoma (EOC)
Exclusion of borderline ovarian tumor, malignant germ cell tumor, sex-cord /stromal tumor, metastatic ovarian carcinoma, and other ovarian malignancy with non-epithelial histologic types
Excluded
31 Pts.: age unknown
62 Pts.: stage unknown
74 Pts.: outcome unknown
2,801 Pts. with EOC
Excluded
1,618 Pts.: stage I-IV EOC
1,183 Pts. with stage I EOC
Excluded
323 Pts. aged ≤ 45 years
860 Pts.: aged >45 years
Excluded
11 Pts.: substage unknown
27 Pts.: inadequate peritoneal staging
285 Pts. enrolled
184 Pts who underwent RS
101 Pts who underwent FSS
Analysis
